# Scale-free networks are rare

**DOI:** 10.1038/s41467-019-08746-5

**Published:** 2019-03-04

**Authors:** Anna D. Broido, Aaron Clauset

**Affiliations:** 1 Department of Applied Mathematics, University of Colorado, 526 UCB, Boulder, CO 80309 USA; 2 Department of Computer Science, University of Colorado, 430 UCB, Boulder, CO 80309 USA; 3 BioFrontiers Institute, University of Colorado, 596 UCB, Boulder, CO 80309 USA; 4 Santa Fe Institute, 1399 Hyde Park Road, Santa Fe, NM 87501 USA

## Abstract

Real-world networks are often claimed to be scale free, meaning that the fraction of nodes with degree *k* follows a power law *k*^−*α*^, a pattern with broad implications for the structure and dynamics of complex systems. However, the universality of scale-free networks remains controversial. Here, we organize different definitions of scale-free networks and construct a severe test of their empirical prevalence using state-of-the-art statistical tools applied to nearly 1000 social, biological, technological, transportation, and information networks. Across these networks, we find robust evidence that strongly scale-free structure is empirically rare, while for most networks, log-normal distributions fit the data as well or better than power laws. Furthermore, social networks are at best weakly scale free, while a handful of technological and biological networks appear strongly scale free. These findings highlight the structural diversity of real-world networks and the need for new theoretical explanations of these non-scale-free patterns.

## Abstract (web summary)

Real-world networks are often said to be ”scale free”, meaning their degree distribution follows a power law. Broido and Clauset perform statistical tests of this claim using a large and diverse corpus of real-world networks, showing that scale-free structure is far from universal.

## Introduction

Networks are a powerful way to represent and study the structure of complex systems. Examples today are plentiful and include social interactions among individuals, protein or gene interactions in biological organisms, communication between digital computers, and various transportation systems. Across scientific domains and classes of networks, it is common to encounter the claim that most or all real-world networks are scale free. The precise details of this claim vary^1–7^, but generally a network is deemed scale free if the fraction of nodes with degree *k* follows a power-law distribution *k*^−*α*^, where *α* > 1. Some versions of this “scale-free hypothesis” have stronger requirements, e.g., requiring 2 < *α* < 3 or that node degrees evolve by the preferential attachment mechanism^8,9^. Other versions make them weaker, e.g., the power law need only hold in the upper tail^10^, it can exhibit an exponential cutoff^11^, or it is merely more plausible than a thin-tailed distribution like an exponential or normal^12^.

The study and use of scale-free networks is widespread throughout network science^1,9,13–15^. Many studies investigate how the presence of scale-free structure shapes dynamics running over a network^6,7,14,16–22^. For example, under the Kuramoto oscillator model, a transition to global synchronization is well-known to occur at a precise threshold *K*_*c*_, whose value depends on the power-law parameter *α* of the degree distribution^23–27^. Scale-free networks are also widely used as a substrate for network-based numerical simulations and experiments, and the study of specific generating mechanisms for scale-free networks has been framed as providing a common basis for understanding all network assembly^3,8,9,28–32^.

The universality of scale-free networks, however, remains controversial. Many studies find support for their ubiquity^4,5,16,17,33–35^, while others challenge it on statistical or theoretical grounds^2,3,10,36–44^. This conflict in perspective has persisted because past work has typically relied upon small, often domain-specific data sets, less rigorous statistical methods, differing definitions of “scale-free” structure, and unclear standards of what counts as evidence for or against the scale-free hypothesis^4–7,16,17,45–48^. Additionally, few studies have performed statistically rigorous comparisons of fitted power-law distributions to alternative, non-scale-free distributions, e.g., the log-normal or stretched exponential, which can imitate a power-law form in realistic sample sizes^49^. These issues raise a natural question of the pervasiveness of strong empirical evidence for scale-free structures in real-world networks.

Central to this debate are the ambiguities induced by the diversity of uses of the term “scale-free network.” The classic definition^1,21,35,37^ states that a network is scale free if its degree distribution Pr(k) has a power law *k*^−*α*^ form. A power law is the only normalizable density function *f*(*k*) for node degrees in a network that is invariant under rescaling, i.e., $f(c\;k) = g(c)f(k)$ for any constant *c*^14^, and thus “free” of a natural scale. For a network’s degree distribution, being scale free implies a power-law pattern, and vice versa. Scale invariance can also refer to non-degree-based aspects of network structure, e.g., its subgraphs may be structurally self-similar^50,51^, and sometimes these networks are also called scale free.

Scale-free networks are commonly discussed in the literature on network assembly mechanisms, particularly in the context of preferential attachment^1,28,29^, in which the probability that a node gains a connection is proportional to its current degree *k*. Although preferential attachment is the most famous mechanism that produces scale-free networks, there exist other mechanisms that can also produce them^13–15^. And, some variations of preferential attachment do not produce power-law degree distributions^35^, although those networks are still sometimes, confusingly, called scale free. Because the shape of a degree distribution imposes only modest constraints on overall network structure^52^, it represents relatively weak evidence when trying to distinguish generating mechanisms^53–56^, even when the distribution’s functional form is clear. However, identifying that form from empirical data can be non-trivial, e.g., because log-normals often fit degree distributions as well or better than power laws^49,56,57^.

Across this broad literature, the term “scale-free network” may mean a precise or approximate statistical pattern in the degree distribution, an emergent behavior in an asymptotic limit, or a property of all networks assembled in part or in whole by a particular family of mechanisms. This imprecision has contributed to the controversy around the scale-free hypothesis.

Here, we focus narrowly on the traditional degree-based definition of a scale-free network, which has the advantage of being directly testable using empirical data. Even within this scope, the definition is often modified by introducing auxiliary hypotheses^58^. For instance, the scale-free pattern may only hold for the largest degrees, implying Pr(*k*) ∝ *k*^−*α*^ for *k* ≥ *k*_min_ > 1, so that the power law governs the distribution’s upper tail, while the lower tail or “body” follows some non-power-law pattern. In other settings, finite-size effects may suppress the frequency of nodes with degrees close to the underlying system’s size, implying Pr(*k*) ∝ *k*^−*α*^*e*^−*λk*^, where *λ* governs the transition between a power law and an exponential cutoff in the extreme upper tail. Or, extreme heterogeneity among degrees may be of primary interest, implying a restriction like 2 < *α* < 3, where the distribution’s mean is finite while its variance is infinite, asymptotically. Finally, the power law may not even be meant to be a good model of the data itself, but rather simply a better model than some alternatives, e.g., an exponential or log-normal distribution, or just a generic stand-in for a “heavy-tailed” distribution, i.e., one that decays more slowly than an exponential.

A consequence of these varied uses of the term scale-free network is that different researchers can use the same term to refer to slightly different concepts, and this ambiguity complicates efforts to empirically evaluate the basic hypothesis. Here, we construct a severe test^58^ of the ubiquity of scale-free networks by applying state-of-the-art statistical methods to a large and diverse corpus of real-world networks. To explicitly cover the variations in how scale-free networks have been defined in the literature, we formalize a set of quantitative criteria that represent differing strengths and types of evidence for scale-free structure in a particular network. This set of criteria unifies the common variations, and their combinations, and allows us to assess different types and degrees of evidence of scale-free degree distributions. For each network data set in the corpus, we estimate the best-fitting power-law model, test its statistical plausibility, and compare it to alternative non-scale-free distributions. We analyze these results collectively, consider how the evidence for scale-free structure varies across domains, and quantitatively evaluate their robustness under several alternative criteria. We conclude with a forward-looking discussion of the empirical relevance of the scale-free hypothesis and offer suggestions for future research on the structure of networks.

## Results

### Preliminaries

**Fig. 1:**
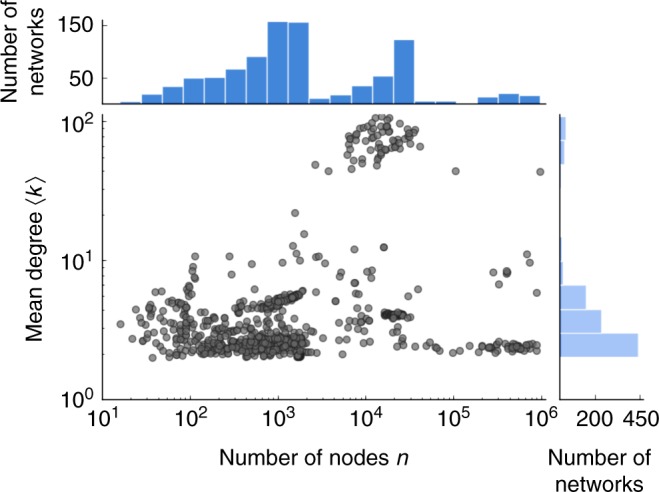
Mean degree $\langle k\rangle$ as a function of the number of nodes *n*. The 928 network data sets in the corpus studied here vary broadly size and density. For data sets with more than one degree sequence (see text), we plot the median of the corresponding set of mean degrees

A key component of our evaluation of the scale-free hypothesis is the use of a large and diverse corpus of real-world networks. This corpus is composed of 928 network data sets drawn from the Index of Complex Networks (ICON), a comprehensive online index of research-quality network data, spanning all fields of science^59^. It includes networks from biological, information, social, technological, and transportation domains that range in size from hundreds to millions of nodes (Fig. 1). These networks also exhibit a wide variety of graph properties, such as being simple, directed, weighted, multiplex, temporal, or bipartite.

The scale-free hypothesis is defined most clearly for simple graphs, which have only one degree distribution. More complicated networks, e.g., a directed, weighted, multiplex network, can have multiple degree distributions, which complicates testing whether it is scale free; we must determine which degree distributions count as evidence and which do not. We address this problem in two ways. First, we apply a sequence of graph transformations that convert a given network data set, defined as a network with multiple graph properties, into a set of simple graphs, each of which can be tested unambiguously for scale-free structure (Supplementary Figs. 1 and 2). In this process, we discard any resulting simple graph that is either too dense or too sparse, under pre-specified thresholds, to be plausibly scale free. (See Supplementary Note 1 for complete details.)

Then, for each simple graph associated with a network data set, we apply standard statistical methods^49^ to identify the best-fitting power law in the degree distribution’s upper tail, evaluate its statistical plausibility using a goodness-of-fit test, and compare it to four alternative distributions fitted to the same part of the upper tail using a likelihood-ratio test. The outputs of these fitting, testing, and comparison procedures for a given simple graph encode in a vector the statistical evidence for its scale-free structure. We then evaluate the set of these vectors for a given network data set under criteria that formalize the different definitions of a scale-free network.

For a given degree distribution, a key step in this process is the selection of a value *k*_min_, above which the degrees are most closely modeled by a scale-free distribution (see Methods). Hence, the fitting procedure truncates non-power-law behavior among low-degree nodes, enabling a more clear evaluation of potentially scale-free patterns in the upper tail. For technical reasons, all model tests and comparisons must then be made only on the degrees *k* ≥ *k*_min_ in the upper tail^49^. Although our primary evaluation uses a normalized likelihood ratio test^60^ that has been specifically shown valid for comparing the distributions considered here^49^, we also present results based on using standard information criteria to compare distributional models^61^.

This approach for evaluating evidence for scale-free structure has several advantages. It provides a systematic procedure applicable to any network data set, and treats every data set equivalently. It provides an evaluation of the scale-free hypothesis over a maximally broad variety of networks, which facilitates the characterization of their empirical ubiquity. And, it provides a means to assess different kinds of evidence for scale-free structure, by combining results from multiple degree distributions, if available in a network data set. The graph-simplification process or the particular evidence criteria used may also introduce biases into the results. We control for these possibilities by considering alternative criteria under multiple robustness analyses.

### Definitions of a scale-free network

**Fig. 2:**
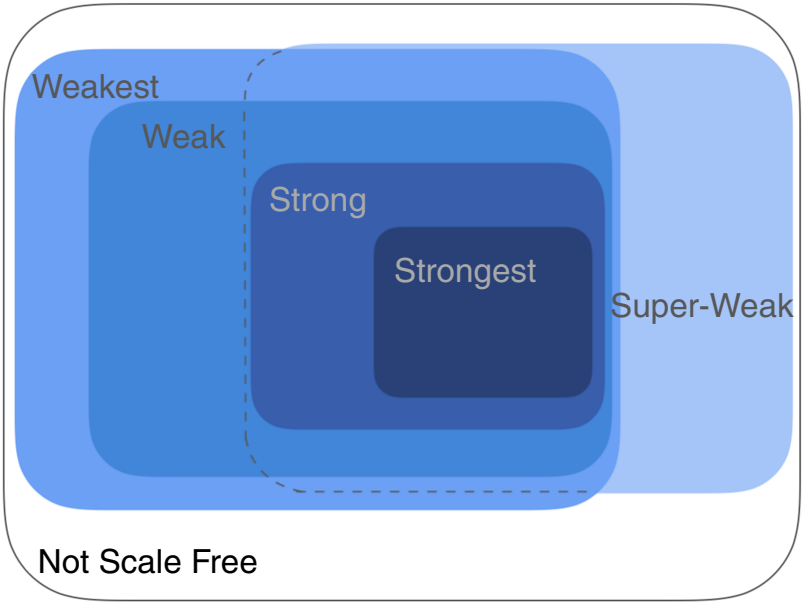
Taxonomy of scale-free network definitions. Super-Weak meaning that a power law is not necessarily a statistically plausible model of a network’s degree distribution but it is less implausible than alternatives; Weakest, meaning a degree distribution that is plausibly power-law distributed; Weak, adds a requirement that the distribution’s scale-free portion cover at least 50 nodes; Strong, adds a requirement that $2 \ < \ \hat \alpha \ < \ 3$ and the Super-Weak constraints; and, Strongest, meaning that almost every associated simple graph can meet the Strong constraints. The Super-Weak overlaps with the Weak definitions and contains the Strong definitions as special cases. Networks that fail to meet any of these criteria are deemed Not Scale Free

The different notions of evidence for scale-free structure found in the literature can be organized into a nearly nested set of categories (Fig. 2) and assessed by applying standard statistical tools to each graph associated with a network data set. Evidence for scale-free structure typically comes in two types: (i) a power-law distribution is not necessarily a good model of the degrees, but it is a relatively better model than alternatives, or (ii) a power law is itself a good model of the degrees.

The first type represents indirect evidence of scale-free structure, because the observed degree distribution is not itself required to be plausibly scale free, only that a scale-free pattern is more believable than some non-scale-free patterns. A network data set that exhibits this kind of evidence is placed into a category called

- Super-Weak: For at least 50% of graphs, no alternative distribution is favored over the power law.

The second type represents direct evidence of scale-free structure, and the various modifications of a purely scale-free pattern can be organized in a set of nested categories that represent increasing levels of evidence:

- Weakest: For at least 50% of graphs, a power-law distribution cannot be rejected (*p* ≥ 0.1).
- Weak: Requirements of Weakest, and the power-law region contains at least 50 nodes (*n*_tail_ ≥ 50).
- Strong: Requirements of Weak and Super-Weak, and $2 \ < \ \hat \alpha \ < \ 3$ for at least 50% of graphs.
- Strongest: Requirements of Strong for at least 90% of graphs, and requirements of Super-Weak for at least 95% of graphs.

The progression from Weakest to Strongest categories represents the addition of more specific properties of the power-law degree distribution, all found in the literature on scale-free networks or distributions. We define a sixth category of networks that includes all networks that do not fall into any of the above categories:

- Not Scale Free: Networks that are neither Super-Weak nor Weakest.

This evaluation scheme is parameterized by the different fractions of simple graphs required by each evidence category. The particular thresholds given above are statistically motivated in order to control for false positives and overfitting, and to provide a consistent treatment across all networks (see Methods). A more permissive parameterization of the scheme is also considered as a robustness check. The above scheme favors finding evidence for scale-free structure in three ways: (i) graphs identified as being too dense or too sparse to be plausibly scale free are excluded from all analyses, (ii) the estimation procedure selects, by choosing *k*_min_, the subset of data in the upper tail that best-fits a power law, and (iii) the comparisons to alternatives are performed only on the data selected by the power law.

### Scaling parameters

**Fig. 3:**
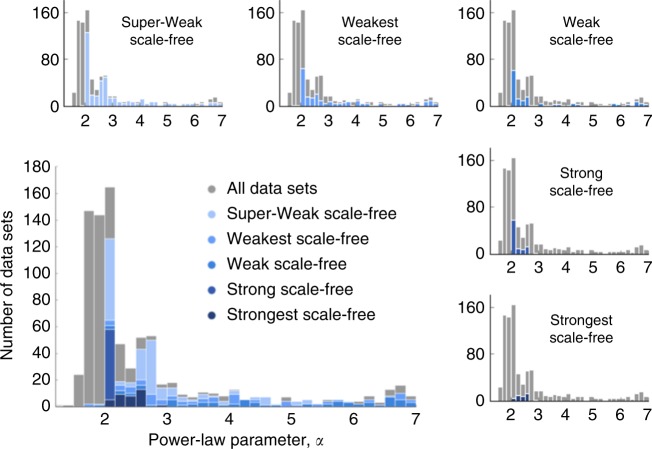
Distribution of $\hat \alpha$ by scale-free evidence category. For networks with more than one degree sequence, the median estimate is used, and for visual clarity the 8% of networks with a median $\hat \alpha \ge 7$ are omitted

Across the corpus, the distribution of median estimated scaling parameters parameters $\hat \alpha$ is concentrated around a value of $\hat \alpha = 2$, but with a long right-tail such that 32% of data sets exhibit $\hat \alpha \ge 3$ (Fig. 3). The range $\alpha \in (2,3)$ is sometimes identified as including the most emblematic of scale-free networks^8,9^, and we find that 39% of network data sets have median estimated parameters in this range. We also find that 34% of network data sets exhibit a median parameter $\hat \alpha \ < \ 2$, which is a relatively unusual value in the scale-free network literature.

Because every network produces some $\hat \alpha$, regardless of the statistical plausibility of the network being scale free, the shape of the distribution of $\hat \alpha$ is not necessarily evidence for or against the ubiquity of scale-free networks. It does, however, enable a check of whether the estimation methods are biased by network size *n*. Comparing $\hat \alpha$ and *n*, we find little evidence of strong systematic bias (*r*^2^ = 0.24, *p* = 1.82 × 10^−13^; Supplementary Fig. 3).

Across the five categories of evidence for scale-free structure, the distribution of median $\hat \alpha$ parameters varies considerably (Fig. 3, insets). For networks that fall into the Super-Weak category, the distribution has a similar breadth as the overall distribution, with a long right-tail and many networks with $\hat \alpha > 3$. Most of the networks with $\hat \alpha \ < \ 2$ are spatial networks, representing mycelial fungal or slime mold growth patterns^62^. However, few of these exhibit even Super-Weak or Weakest evidence of scale-free structure, indicating that they are not particularly plausible scale-free networks. Among the Weakest and Weak categories, the distribution of median $\hat \alpha$ remains broad, with a substantial fraction exhibiting $\hat \alpha > 3$. The Strong and Strongest categories require that $\hat \alpha \in (2,3)$, and the few network data sets in these categories are somewhat concentrated near $\hat \alpha = 2$.

### Alternative distributions

Independent of whether the power-law model is a statistically good model of a network’s degree sequence, it may nevertheless be a better model than non-power-law alternatives.

Across the corpus, likelihood ratio tests find only modest support for the power-law distribution over four alternatives (Table 1). In fact, the exponential distribution, which exhibits a thin tail and relatively low variance, is favored over the power law (41%) more often than vice-versa (33%). This outcome accords with the broad distribution of scaling parameters, as when *α* > 3 (32% of data sets; Fig. 3), the degree distribution must have a relatively thin tail.

**Table 1:** Comparison of scale-free and alternative distributions

		Test outcome		
Alternative	*p*(*x*)∝*f*(*x*)	*M*_P *L*_	Inconclusive	*M*_A *lt*_
Exponential	*e* ^− *λx*^	33%	26%	41%
Log-normal	$\frac{1}{x}\;e^{ - \frac{{\left( {logx - \mu } \right)^2}}{{2\sigma ^2}}}$	12%	40%	48%
Weibull	$e^{ - \left( {\frac{x}{b}} \right)^a}$	33%	20%	47%
Power law with cutoff	*x* ^− *α*^ *e* ^− *λx*^	–	44%	56%

The percentage of network data sets that favor the power-law model *M*_P*L*_, alternative model *M*_A*lt*_, or neither, under a likelihood-ratio test, along with the form of the alternative distribution *f*(*x*)

The log-normal is a broad distribution that can exhibit heavy tails, but which is nevertheless not scale free. Empirically, the log-normal is favored more than three times as often (48%) over the power law, as vice versa (12%), and the comparison is inconclusive in a large number of cases (40%). In other words, the log-normal is at least as good a fit as the power law for the vast majority of degree distributions (88%), suggesting that many previously identified scale-free networks may in fact be log-normal networks.

The Weibull or stretched exponential distribution can produce thin or heavy tails, and is a generalization of the exponential distribution. Compared to the power law, the Weibull is more often the better statistical model (47%) than vice versa (33%). Finally, the power-law distribution with an exponential cut-off requires special consideration, as it contains the pure power-law model as a special case. As a result, the likelihood of the power law can never exceed that of the cutoff model, and the interesting outcome is the degree to which the test is inconclusive between the two. In this case, a majority of networks (56%) favor the power law with cutoff model, indicating that finite-size effects may be common.

The above findings are corroborated by replacing the likelihood ratio test with information criteria to perform the model comparisons, which yield qualitatively similar conclusions (Supplementary Table II).

### Assessing the scale-free hypothesis

Given the results of fitting, testing, and comparing the power-law distribution across networks, we now classify each according to the six categories described above.

**Fig. 4:**
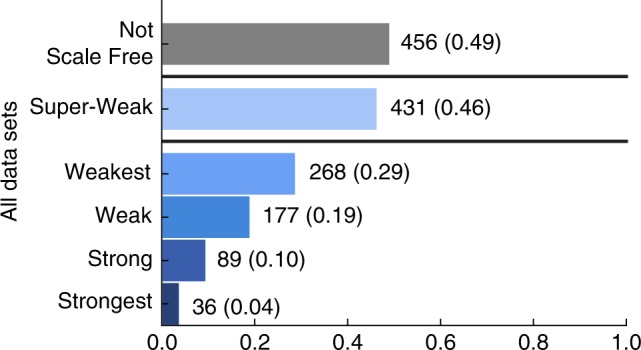
Proportion of networks by scale-free evidence category. Bars separate the Super-Weak category from the nested definitions, and from the Not Scale Free category, defined as networks that are neither Weakest or Super-Weak

Across the corpus, fully 49% of networks fall into the Not Scale Free category (Fig. 4). Slightly less than half (46%) fall into the Super-Weak category, in which a scale-free pattern among the degrees is not necessarily statistically plausible itself, but remains no less plausible than alternative distributions. The Weakest and Weak categories represent networks in which the power-law distribution is at least a statistically plausible model of the networks’ degree distributions. In the Weak case, this power-law scaling covers at least 50 nodes, a relatively modest requirement. These two categories account for only 29 and 19% of networks, respectively, indicating that it is uncommon for a network to exhibit direct statistical evidence of scale-free degree distributions.

Finally, only 10 and 4% of network data sets can be classified as belonging to the Strong or Strongest categories, respectively, in which the power-law distribution is not only statistically plausible, but the exponent falls within the special $\alpha \in (2,3)$ range and the power law is a better model of the degrees than alternatives. Taken together, these results indicate that genuinely scale-free networks are far less common than suggested by the literature, and that scale-free structure is not an empirically universal pattern.

**Fig. 5:**
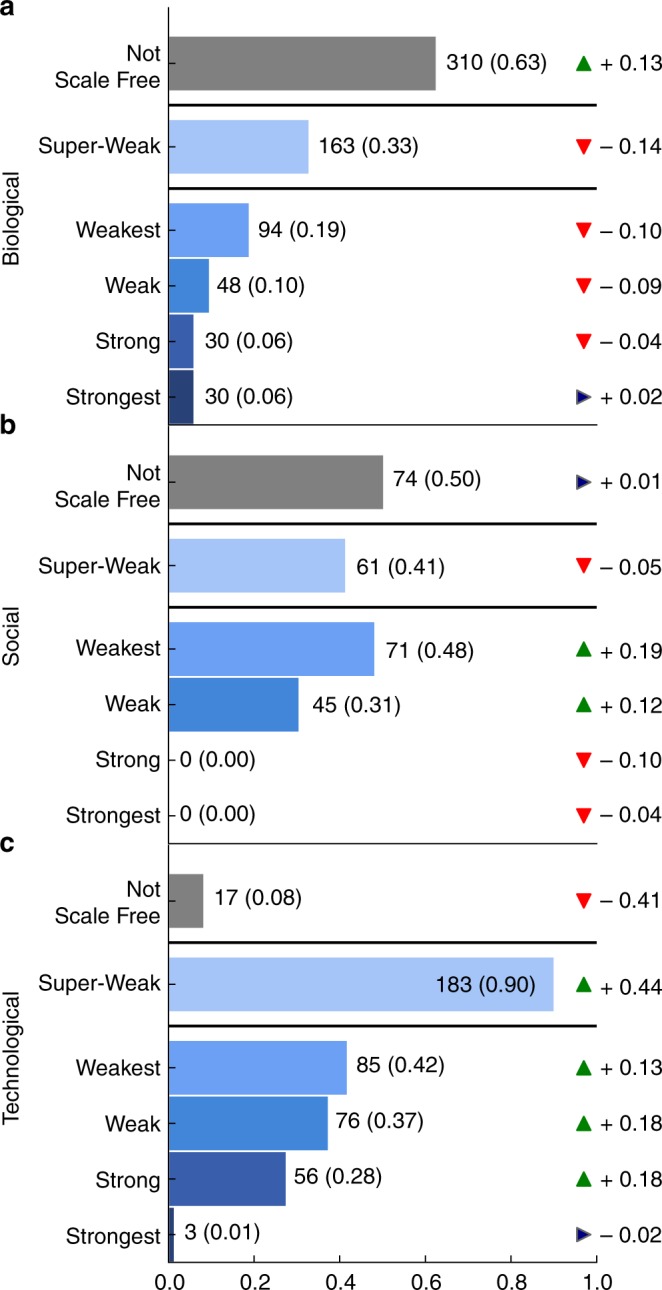
Proportion of networks by scale-free evidence category and by domain. **a** Biological networks, **b** social networks, and **c** technological networks. Tickers show change in percent from the pattern in all of the data sets

The balance of evidence for or against scale-free structure does vary by network domain (Fig. 5). These variations provide a means to check the robustness of our results, and can inform future efforts to develop new structural mechanisms. We focus our domain-specific analysis on networks from biological, social, and technological sources (91% of the corpus).

Among biological networks, a majority lack any direct or indirect evidence of scale-free structure (63% Not Scale Free; Fig. 5a), in agreement with past work on smaller corpora of biological networks^42^. The aforementioned fungal networks represent a large share of these Not Scale Free networks, but this group also includes some protein interaction networks and some food webs. Among the remaining networks, one third exhibit only indirect evidence (33% Super-Weak), and a modest fraction exhibit the weakest form of direct evidence (19% Weakest). This latter group includes cat and rat brain connectomes. Compared to the corpus as a whole, biological networks are slightly more likely to exhibit the strongest level of direct evidence of scale-free structure (6% Strongest), and these are primarily metabolic networks.

We note that the fungal networks comprise 28% of the corpus and our analysis places 100% of them in the Not Scale Free category. Given their spatially embedded nature, it could be argued that these networks were unlikely to be scale-free in the first place. Because we know a posteriori that these networks are Not Scale Free, omitting them will necessarily increase the fraction of networks in at least some of the other categories. We find that these increases occur primarily in the weaker evidence categories: 5% of non-fungal networks fall into the Strongest category (up from 4%), 13% in Strong (from 10%), 27% in Weak (from 19%), 40% in Weakest (from 29%), and 65% Super-Weak (from 46%). Hence, the qualitative conclusions from our primary analysis are robust to the inclusion of this particular subset of networks.

In contrast, social networks present a different picture. Like the corpus overall, half of social networks lack any direct or indirect evidence of scale-free structure (50% Not Scale Free; Fig. 5b), while indirect evidence is slightly less prevalent (41% Super-Weak). The former group includes the Facebook100 online social networks, and the latter includes many Norwegian board of director networks.

However, among the categories representing direct evidence of scale-free structure, more networks fall into the Weakest (48%) and Weak (31%) categories, but not a single network falls into the Strong or Strongest categories. Hence social networks are at best only weakly scale free, and even in cases where the power-law distribution is plausible, non-scale-free distributions are often a better description of the data. The social networks exhibiting weak evidence include many scientific collaboration networks and roughly half of the Norwegian board of director networks.

Technological networks exhibit the smallest share of networks for which there is no evidence, direct or indirect, of scale-free structure (8% Not Scale Free; Fig. 5c), and the largest share exhibiting indirect evidence (90% Super-Weak). The former group includes some digital circuit networks and various water distribution networks. Among the categories representing direct evidence, less than half exhibit the weakest form of direct evidence (42% Weakest). This group includes roughly half of CAIDA’s networks of autonomous systems, several digital circuit networks, and several peer-to-peer networks. In contrast to biological or social networks, however, technological networks exhibit a modest fraction with strong direct evidence of scale-free structure (28% Strong). Networks in this category include the other half of the CAIDA graphs. But, almost none of the technological networks exhibit the strongest level of direct evidence (1% Strongest).

Transportation networks do not represent a large enough fraction of the corpus for a similar statistical analysis, but do offer some useful insights for future work. Most of these networks exhibit little evidence of scale-free structure. For example, all three airport networks and 46 of 49 road networks fall into the Not Scale Free category, while two of the remaining three road networks fall into the Weak category and one into Super-Weak. All of the subway networks fall into the Super-Weak category, and nearly all fall into the Weakest category. These results suggest that scale-free networks may represent poor models of many transportation systems.

### Robustness analysis

In order to assess the dependence of these results on the evaluation scheme itself, we conduct a series of robustness tests.

Specifically, we test whether the above results hold qualitatively when (i) we consider only network data sets that are naturally simple (unweighted, undirected, monoplex, and no multi-edges); (ii) we remove the power-law with cutoff from the set of alternative distributions; (iii) we lower the percentage thresholds for all categories to allow admission if any one constituent simple graph satisfies the requirements; and (iv) we analyze the scaling behavior of the degree distribution’s first and second moment ratio. Details for each of these tests, and two others, are given in Supplementary Note 5. We also test whether the evaluation scheme correctly classifies four different types of synthetic networks with known structure, both scale free and non-scale free. Details and results for these tests are given in Supplementary Note 6.

The first test evaluates whether the extension of the scale-free hypothesis to non-simple networks and the corresponding graph-simplification procedure biases the results. The second evaluates whether the presence of finite-size effects drives the lack of evidence for scale-free distributions. Applied to the corpus, each test produces qualitatively similar results as the primary evaluation scheme (see Supplementary Note 5, and Supplementary Fig. 4), indicating that the lack of empirical evidence for scale-free networks is not driven by these particular choices in the evaluation scheme itself.

The third considers a “most permissive” parameterization, which evaluates the impact of our requirements that a minimum percentage of degree sequences satisfy the constraints of a category. Under this test, we specifically examine how the evidence changes if we instead require that only one degree sequence satisfies the given requirements. That is, this test lowers the threshold for each category to be maximally permissive: if scale-free structure exists in any associated degree sequence, the network data set is counted as falling into the corresponding category.

**Fig. 6:**
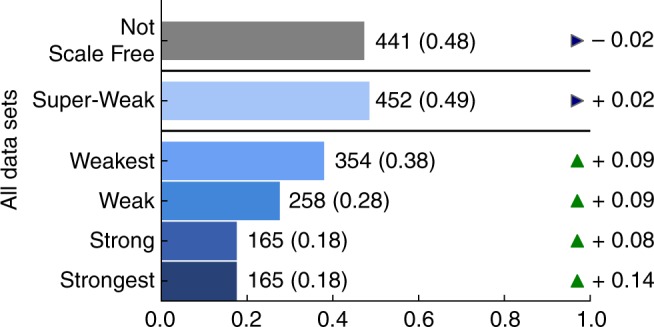
Proportions of networks in each scale-free evidence category with removed degree percentage requirements

Under this modification, the Strong and Strongest categories become equivalent, and 18% of network data sets fall into this combined category (Fig. 6). We note that under this modified evaluation, synthetic directed networks assembled by preferential attachment should and do fall into the Strongest category of evidence. The most permissive category, Super-Weak, only changes slightly from 46 to 49%. And finally, performing this test on only the directed networks within the corpus produces similar results (see Supplementary Note 5 and Supplementary Fig. 5). These tests demonstrate that the percentage requirements used in the category definitions of the primary evaluation scheme are not overly restrictive, and our qualitative conclusions are robust to variations in the precise thresholds the evaluation uses.

**Fig. 7:**
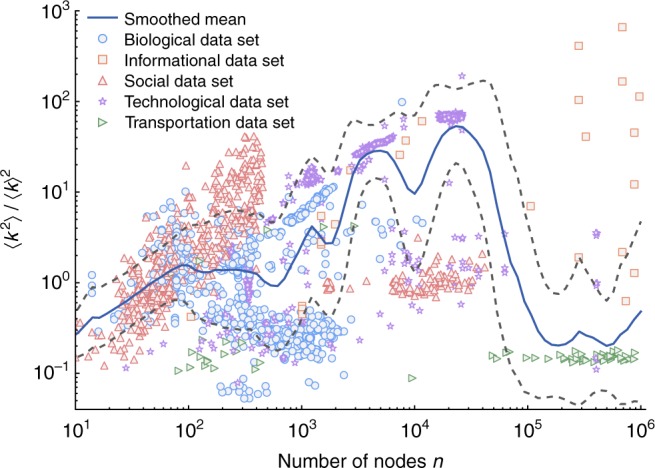
Moment ratio scaling. For 3662 degree sequences, the empirical ratio of the second to first moments $\langle k^2\rangle /\langle k\rangle ^2$ as a function of network size *n*, showing substantial variation across networks and domains, little evidence of the divergence pattern expected for scale-free distributions, and perhaps a roughly sublinear scaling relationship (smoothed mean via exponential kernel, with smoothed standard deviations)

The fourth test provides a model-independent evaluation of a key prediction of the scale-free hypothesis. Scale-free distributions are mathematically unusual because only the moments $\langle k^m\rangle$ for *m* < *α* – 1 are finite, and all higher moments diverge^14^, asymptotically. Hence, in the most widely analyzed range of $\alpha \in (2,3)$ for scale-free networks, the moment ratio $\langle k^2\rangle /\langle k\rangle ^2$ diverges as the network size *n* increases. This behavior underpins the practical relevance of many theoretical analyses of scale-free networks. Of course, diverging moments cannot be identified from finite-sized networks, and no real-world network can validate this prediction of the scale-free hypothesis. However, if most networks are scale free in this way, the scaling behavior of their moment ratios should exhibit a strongly diverging trend. Across the corpus as a whole, we find little evidence of a general pattern of diverging moment ratios (Fig. 7). Instead, we find enormous variation in ratios across networks, domains, and scales, such that networks with $10^2 \le n \le 10^3$ often have larger ratios than networks several orders of magnitude larger, and even those moments that do appear to increase with *n* do not increase fast enough to be consistent with scale-free behavior (Supplementary Fig. 8). We leave a more detailed investigation of these variations for future work.

Overall, the results of these tests corroborate our primary findings of relatively little empirical evidence for the ubiquity of scale-free networks, and suggest that empirical degree distributions exhibit a richer variety of patterns, many of which are lower variance, than predicted by the scale-free hypothesis.

## Discussion

By evaluating the degree distributions of nearly 1000 real-world networks from a wide range of scientific domains, we find that scale-free networks are not ubiquitous. Fewer than 36 networks (4%) exhibit the strongest level of evidence for scale-free structure, in which every degree distribution associated with a network is convincingly scale free. Only 29% of networks exhibit the weakest form, in which a power law is simply a statistically plausible model of some portion of the degree distribution’s upper tail. And, for 46% of networks, the power-law form is not necessarily itself a good model of the degree distribution, but is simply a statistically better model than alternatives. Nearly half (49%) of networks show no evidence, direct or indirect, of scale-free structure, and in 88% of networks, a log-normal fits the degree distribution as well as or better than a power law. These results demonstrate that scale-free networks are not a ubiquitous phenomenon, and suggest that their use as a starting point for modeling and analyzing the structure of real networks is not empirically well grounded.

Across different scientific domains, the evidence for scale-free structure is generally weak, but varies somewhat in interesting ways. These differences provide hints as to where scale-free structure may genuinely occur. For instance, our evidence indicates that scale-free patterns are more likely to be found in certain kinds of biological and technological networks. These findings corroborate theoretical work on domain-specific mechanisms for generating scale-free structure, e.g., in biological networks via the well-established duplication-mutation model for molecular networks^3,30,54^ or in certain kinds of technological networks via highly optimized tolerance^13,63^.

In contrast, we find that social networks are at best weakly scale free, and although a power-law distribution can be a statistically plausible model for these networks, it is often not a better model than a non-scale-free distribution. Class imbalance in the corpus precludes broad conclusions about the prevalence of scale-free structure in information or transportation networks. However, the few of these in the corpus provide little indication that they would exhibit strongly different structural patterns than the better represented domains.

The variation of evidence across social, biological, and technological domains (Fig. 5) is consistent with a general conclusion that no single universal mechanism explains the wide diversity of degree structures found in real-world networks. The failure to find broad evidence for scale-free patterns in the degree distributions of networks indicates that much remains unknown about how network structure varies across different domains^64^ and what kinds of structural patterns are common across them. We look forward to new investigations of statistical differences and commonalities, which seem likely to generate new insights about the structure of complex systems.

The statistical evaluation here considers only the degree distributions of networks, and hence says relatively little about other structural patterns or the underlying processes that govern the form of any particular network. However, the finding that scale-free networks are empirically uncommon does imply a generally limited role for any mechanism that necessarily produces power-law degree distributions^9,15,32,56^, especially in domains where the evidence for strongly scale-free networks is weak, e.g., social networks. The mechanisms that govern the shape of a particular network generally cannot be determined from a static network’s degree distribution alone, as it is both a weak constraint on network structure^52^ and a weak discriminator between mechanisms^54^. For some networks, there is strong evidence that mechanisms like preferential attachment apply, e.g., scientific citation networks^28,29,55,56^. However, the results described here imply that if such mechanisms apply more broadly, they are heavily modified or even dominated by other, perhaps domain-specific mechanisms. A claim that some network is scale free should thus be established using a severe statistical test^58^ that goes beyond static degree distributions.

In theoretical network science, assuming a power law for a random graph’s degree distribution can simplify mathematical analyses, and a power law can be a useful conceptual model for building intuition about the impact of extreme degree heterogeneity. And, for some types of calculations, e.g., the location of the epidemic threshold, scale-free networks can be useful models, even when real-world degree distributions are simply heavy tailed, rather than scale free^65–67^. On the other hand, if a mathematical result depends strongly on the asymptotic behavior of a scale-free degree distribution, the results’ practical relevance will necessarily depend on the empirical prevalence of scale-free structures, which we show to be uncommon or rare, depending on the kind of scale-free structure of interest. Mathematical results based on extreme degree heterogeneity may, in fact, have more narrow applicability than previously believed, given the lack of evidence that empirical moment ratios diverge as quickly as those results typically assume (Fig. 7 and Supplementary Fig. 8).

The structural diversity of real-world networks uncovered here presents both a puzzle and an opportunity. The strong focus in the scientific literature on explaining and exploiting scale-free patterns has meant relatively less is known about mechanisms that produce non-scale-free structural patterns, e.g., those with degree distributions better fitted by a log-normal. Two important directions of future work will be the development and validation of novel mechanisms for generating more realistic degree structure in networks, and novel statistical techniques for identifying or untangling them given empirical data. Similarly, theoretical results concerning the behavior of dynamical processes running on top of networks, including spreading processes like epidemiological models, social influence models, or models of synchronization, may need to be reassessed in light of the genuine structural diversity of real-world networks.

The statistical methods and evidence categories developed and used in our evaluation of the scale-free hypothesis provide a quantitatively rigorous means by which to assess the degree to which some network exhibits scale-free structure. Their application to a novel network data set should enable future researchers to determine whether assuming scale-free structure is empirically justified.

Furthermore, large corpora of real-world networks, like the one used here, represent a powerful, data-driven resource by which to investigate the structural variability of real-world networks^64^. Such corpora could be used to evaluate the empirical status of many other broad claims in the networks literature, including the tendency of social networks to exhibit high clustering coefficients and positive degree assortativity^68^, the prevalence of the small-world phenomena^69^, the prevalence of “rich clubs” in networks^70^, the ubiquity of community^71^ or hierarchical structure^72^, and the existence of “super-families” of networks^73^. We look forward to these investigations and the new insights they will bring to our understanding of the structure and function of networks.

## Methods

### Network data sets

Network data sets were obtained through the ICON^59^, an online index of real-world network data sets from all domains of science. The composition of the corpus is roughly half biological networks, a third social or technological networks, and a sixth information or transportation networks (Supplementary Table 1). The 928 networks included span five orders of magnitude in size, are generally sparse with a mean degree of $\langle k\rangle \approx 3$ (Fig. 1), and possess a range of graph properties, e.g., simple, directed, weighted, multiplex, temporal, or bipartite.

Prior to analysis, each network data set is transformed into one or more graphs, whose degree sequences can be unambiguously tested for a scale-free pattern (for example, Supplementary Fig. 1). For each non-simple graph property of a network, a specific transformation is applied that increases the number of graphs in the data set while removing the given graph property. Full details of this process are given in Supplementary Note 1, and Supplementary Fig. 2. Complicated network data sets can produce a combinatoric number of simple graphs under this process. Treating every simplified degree sequence independently could lead to skewed results, e.g., if a few non-scale-free data sets account for a large fraction of the total extracted simple graphs. To avoid this bias, results are reported at the level of network data sets. Additionally, we require that simplified graphs are neither too sparse nor too dense to be potentially scale free and thus retain for analysis only simplified graphs with mean degree $2 \ < \ \langle k\rangle \ < \ \sqrt n$.

Simplifying the 928 network data sets produced 18,448 simple graphs, of which 14,415 were excluded for being too sparse and 371 excluded for being too dense (about 80.4% of derived simple graphs). Results in the main text are reported only in terms of the remaining 3662 simple graphs (about 3.9 per network data set). Of the 928 network data sets, 735 (79%) produced no graphs that were excluded for being too sparse. More than 90% of graphs excluded for being too sparse were produced by simplifying three network data sets (<1% of the corpus). Similarly, 874 (94%) of the network data sets produced no graphs that were excluded for being too dense. More than 70% of graphs excluded for being too dense were produced by simplifying three network data sets. Finally, 782 (84%) of the data sets generated at most three degree sequences prior to applying the too-sparse and too-dense filters. Hence, the vast majority of data sets were uninvolved in the production of many excluded graphs.

### Modeling degree distributions

For the degree sequence $\{ k_i\} = k_1,k_2, \ldots ,k_n$ of a given network data set, we estimate the best-fitting power-law distribution of the form

1 $${\mathrm{Pr}}(k) = C\;k^{ - \alpha }\quad \quad \alpha \ > \ 1,\quad k \ge k_{{\mathrm{min}}} \ge 1,$$

where *α* is the scaling exponent, *C* is the normalization constant, and *k* is integer valued. This specification models only the distribution’s upper tail, i.e., degree values *k* ≥ *k*_min_, and discards data from any non-power-law portion in the lower distribution.

Fitting this model to an empirical degree sequence requires first choosing the location $\hat k_{\mathrm{min}}$ at which the upper tail begins, and then estimating the scaling exponent $\hat \alpha$ on the truncated data $k \ge \hat k_{\mathrm{min}}$. Because the choice of *k*_min_ changes the sample size, it cannot be directly estimated using likelihood or Bayesian techniques. Here, the standard KS-minimization approach is used to choose $\hat k_{\mathrm{min}}$ and the discrete maximum likelihood estimator is used to choose $\hat \alpha$^49^. Technical details of the estimation procedure are given in Supplementary Note 2.

Fitting the power-law distribution always returns some parameters $\hat \theta = (\hat k_{{\mathrm{m}}in},\hat \alpha )$. However, parameters alone give no indication of the quality of the fitted model. A standard goodness-of-fit test is used to assess the statistical plausibility of the fitted model, which returns a standard *p*-value (see Supplementary Note 2). Following standard practice in this setting^49^, if *p* ≥ 0.1, then the degree sequence is deemed plausibly scale free, while if *p* < 0.1, the scale-free hypothesis is rejected. Hence, if the underlying data generating process is indeed scale free, this test has a false negative rate of 0.1. The results of this test provide direct evidence for or against a network exhibiting scale-free structure.

Each power-law model $\hat \theta$ is compared to four non-scale-free alternative models, estimated via maximum likelihood on the same degrees $k \ge \hat k_{\mathrm{min}}$, using a standard Vuong normalized likelihood ratio test (LRT)^49,60^ (see Supplementary Notes 3, 4). The restriction to $k \ge \hat k_{\mathrm{min}}$ is necessary to make the model likelihoods directly comparable, and slightly biases the test in favor of the power law, as the best choice of $\hat k_{\mathrm{min}}$ for an alternative may not be the same as the best choice for the power law^49^. The results of this test provide indirect evidence about the scale-free hypothesis, as a power-law model can be favored over some alternative even if the power law itself is not a statistically plausible model of the data. The non-scale free alternatives used here are the (i) exponential, (ii) log-normal, (iii) power-law with exponential cutoff, and (iv) stretched exponential or Weibull distributions (Table 1), all of which have been used previously as models of degree distributions^74–78^, and for which the validity of the LRT used here has specifically been previously established^49^. Results from an alternative comparison based on information criteria^61^ are given in Supplementary Table II and in Supplementary Figs. 6 and 7.

The fitted power law and each alternative are compared using a likelihood ratio test (see Supplementary Note 4), with the test statistic ${\cal R} = {\cal L}_{{\mathrm{PL}}} - {\cal L}_{{\mathrm{Alt}}},$ where ${\cal L}_{{\mathrm{PL}}}$ is the log-likelihood of the power-law model and ${\cal L}_{{\mathrm{Alt}}}$ is the log-likelihood of a particular alternative model. The sign of ${\cal R}$ indicates which model is a better fit to the data: the power law $\left( {{\cal R} \ > \ 0} \right)$, the alternative $({\cal R} \ < \ 0)$, or neither $\left( {{\cal R} = 0} \right)$.

The test statistic ${\cal R}$ is derived from data, meaning that it is itself a random variable subject to statistical fluctuations^49,60^. As a result, the sign of ${\cal R}$ is meaningful only if its magnitude $|{\cal R}|$ is statistically distinguishable from $0$. This determination is made by a standard two-tailed test against a null hypothesis of ${\cal R} = 0$, which yields a standard *p*-value. If *p* ≥ 0.1, then $|{\cal R}|$ is statistically indistinguishable from 0 and neither model is a better explanation of the data than the other. If *p* < 0.1, then the data provide a clear conclusion in favor of one model or the other, depending on the sign of ${\cal R}$. This threshold sets the false positive rate for the alternative distribution at 0.05. Corrections for multiple tests, e.g., a family-wise error rate method like Bonferroni or a false discovery correction like Benjamini-Hochberg, are not employed. Such corrections would simply lower the obtained *p*-values without changing the overall conclusions, while introducing additional assumptions into the analysis.

To report results at the level of a network data set, we apply the LRTs to all the associated simple graphs and then aggregate the results. For each alternative distribution, we count the number of simple graphs associated with a particular network data set in which the outcome favored the alternative, favored the power law, or had an inconclusive result. Normalizing these counts across outcome categories provides a continuous measure of the relative evidence that the data set falls into each of category.

### Parameters for defining scale-free network

Threshold parameters for the primary evaluation criteria were selected to balance false positive and false negative rates, and to provide a consistent evaluation of evidence independent of the associated graph properties or source of data. For the Super-Weak and Weakest categories, a threshold of 50% ensures that the given property is present in a majority of simple graphs associated with a network data set. For the Weak category, a threshold of at least 50 nodes covered by the best-fitting power law in the upper tail follows standard practices^49^ to reduce the likelihood of false positive errors due to low statistical power. For the Strong category, $\alpha \in (2,3)$ covers the full parameter range for which scale-free distributions have an infinite second moment but a finite first moment. For the Strongest category, the thresholds of 90% for the goodness-of-fit test and 95% for likelihood ratio tests against alternatives match the expected error rates for both tests under the null hypothesis. If every graph associated with a network data set is scale free, the goodness-of-fit test is expected to incorrectly reject the power-law model 0.1 of the time, and the likelihood ratio test will falsely favor the alternative 0.05 of the time. In the “most permissive” parameterization of the scheme (see Supplementary Note 5), we relax the threshold requirements so that if at least one graph meets the given criteria, the network is placed in this category. In this permissive parameterization, a directed network with a power-law distribution in the in-degrees should be and is classified as Strongest.

For specific networks, domain knowledge may suggest that some degree sequences are potentially scale free while others are likely not. A non-uniform weighting scheme on the set of associated degree sequences would allow such prior knowledge to be incorporated in a Bayesian fashion. However, no fixed non-uniform scheme can apply universally correctly to networks as different as, for example, directed trade networks, directed social networks, and directed biological networks. To provide a consistent treatment across all networks, regardless of their properties or source, we employ an uninformative (uniform) prior, which assigns equal weight to each associated degree sequence. In future work on specific subgroups of networks, a domain-specific weight scheme could be used with the evaluation criteria described here.

### Results for synthetic networks

The accuracy of the fitting, comparing, and testing methods, and the overall evaluation scheme itself, were evaluated using four classes of synthetic data with known structure. Three of these generated networks that contain power-law degree distributions: a directed version of preferential attachment^79^, a directed vertex copy model^21^, and a simple temporal power-law random graph. One generated networks that do not: simple Erdös-Rényi random graphs. Applied to synthetic networks generated by these models, our evaluation scheme correctly classified each of the synthetic network data sets according to the scale-free categories suitable for their generating parameters (see Supplementary Note 6).

## Supplementary information

Supplementary Information

## Data Availability

The network data sets used are available via https://icon.colorado.edu. Code for graph-simplification functions and power-law evaluations, and data for replication are available at https://github.com/adbroido/SFAnalysis.
