# Supplementary material for: Scale-free networks are rare
Source: Nat Commun. 2019 Mar 4;10:1017. doi: 10.1038/s41467-019-08746-5 (PMC6399239; doi:10.1038/s41467-019-08746-5)
Supplement: Supplementary file 1 — Supplementary Information [file 41467_2019_8746_MOESM1_ESM.pdf]

Supplementary Information: Scale-free networks are rare

Anna D. Broido and Aaron Clauset

SUPPLEMENTARY METHODS

Supplementary Note 1. Simplifying Network Data Sets

Our corpus of real-world networks includes both simple graphs and networks with various combinations of directed, weighted, bipartite, multigraph, temporal, and multiplex properties (Supplementary Table I). For each property, there can be multiple ways to extract a degree sequence, and in some cases, extracting a degree sequence requires making a choice. To resolve these ambiguities, we developed a set of graph simplification functions, which are applied in a sequence that depends only on the graph properties of the input (Supplementary Figures 1, 2).The purpose of this graph simplification algorithm is to provide an objective and consistent set of rules by which to extract a set of degree sequences from any given network data set. This approach thus removes researcher subjectivity in deciding which data set to include or exclude in any evaluation of the scale-free hypothesis, and ensures that the evaluation is as broad as possible. For completeness, we describe these specific pathways, and give counts of how many network data sets in our corpus followed each pathway.

Domain	Number	(Prop.)	Multiplex	Bipartite	Multigraph	Weighted	Directed	Simple
Bio.	495	(0.53)	273	41	378	29	37	39
Info.	16	(0.02)	0	0	4	0	5	7
Social	147	(0.16)	7	0	6	8	0	129
Tech.	203	(0.22)	122	0	3	1	195	5
Trans.	67	(0.07)	48	0	65	3	2	0
Total	928	(1.00)	450	41	456	41	239	180

Supplementary Table I. Number of network data sets, and proportion of our network corpus, in each of five domains, under the taxonomy given by the *Index of Complex Networks* [1].

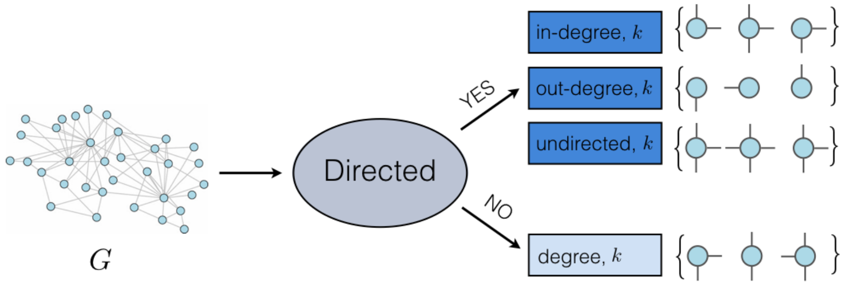

Supplementary Figure 1. A graph simplification function, which takes as input a network G. In this case, if G is directed, the function returns three degree sequences: the in-degrees, out-degrees, and undirected degrees, while if G is undirected, it returns the degree sequence. Supplementary Note 1 contains complete details.

At each stage in our processing we remove one graph property, making the network simpler and never adding properties. Repeating this process for each property in succession converts a network data set into a set of simple graphs. Some networks are processed into a large number of simple graphs, due to the combinatoric effect of certain graph properties. To moderate the amount of combinatoric blowup, we treat weighted graphs differently depending on whether or not they have any multiplex, bipartite, or multigraph properties. Multiplex networks include temporal networks as a special case; many of these have a large number of layers, each of which can generate many simple graphs (see below).

If a weighted graph has any of the aforementioned properties, we simply ignore the edge weights and process the remaining properties. If not, however, the data set is replaced with three unweighted graphs as follows. The goal of this transformation is to replace a potentially dense weighted graph, e.g., a data set representing pairwise similarity scores or correlations, with a set of unweighted graphs that are relatively sparse. To carry out this conversion, we choose thresholds intended to produce sparse graphs that are not so sparse as to be too strongly disconnected to be potentially scale free. Toward this end, we identify and then apply three thresholds to the edge weights, so that the resulting unweighted graphs have a mean degree $\langle k\rangle = \{2, n^{1/4}, \sqrt{n}\}$. These threshold values are determined by the empirical edge weight distribution of the graph, and correspond to choosing the $m = \{n, (1/2)n^{5/4}, (1/2)n^{3/2}\}$

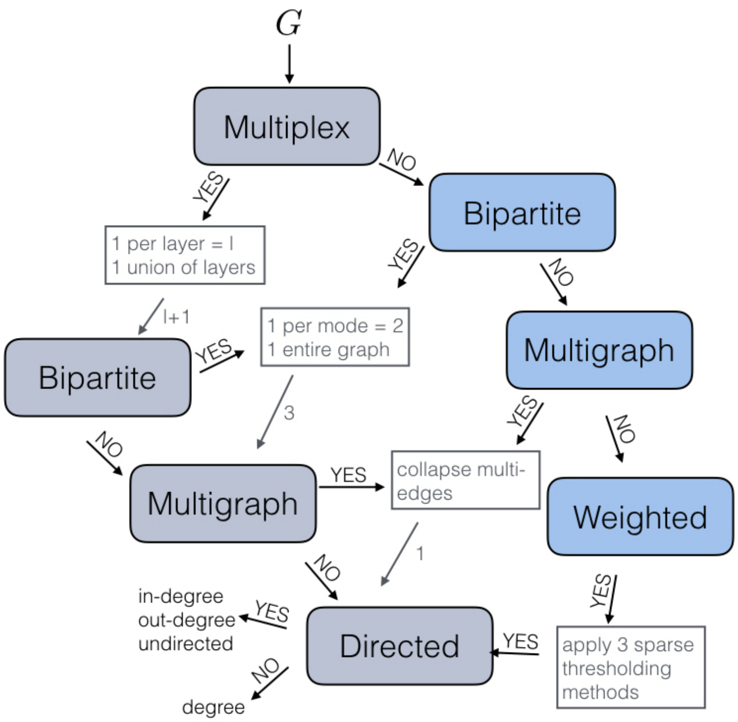

Supplementary Figure 2. Flowchart describing the path from network data set to degree sequence(s). Each step removes a layer from the properties. The gray path is for multiplex, bipartite, or multigraph networks, while the blue is for weighted networks without these properties. Details in text.

largest-weight edges, respectively. The lower value of $\langle k \rangle$ or m produces a very sparse graph, retaining primarily the largest-weight edges, but not so sparse as to be likely strongly disconnected. The upper value produces a more well connected network, retaining all but the smallest-weight edges, but not so dense that the degree distribution is trivial. The middle value splits the difference between these. Our corpus contains only 8 weighted networks and 6 weighted directed networks for 14 total weighted networks, meaning that these networks represent a modest share (2%) of the corpus.

Multiplex and temporal network data sets are composed of T "layers," each of which is a network itself. The multiplex network is replaced by a set of $T+1$ graphs, one for each layer and one for the union of edges and nodes across all layers. In this way, the multiplex or temporal property is removed, and the original data set replaced with a set of graphs. Each graph in this set is then further processed to remove any remaining non-simple properties. A bipartite graph is replaced with three graphs: one each for the "A-mode" projection, "B-mode" projection, and original bipartite graph. If present, multi-edges are collapsed and weights discarded.

As a final step, directed graphs are replaced by three degree sequences: one for the in-degrees, one for the out-degrees, and one for the total degrees; undirected graphs are replaced with their single degree sequence. The results of this sequential processing is a set of degree sequences that, as a group, represent the original network. Our corpus contains 5 pure multiplex networks, 315 multiplex multigraphs, and 130 multiplex directed networks, which yields 450 total multiplex networks.

Network data sets that are bipartite and not multiplex are first replaced with three graphs: one for the "A-mode" project, one for the "B-mode" projection, and one for the original bipartite graph. Each of these graphs is then processed starting from just after the bipartite step described above in the multiplex or temporal network processing pathway. In our corpus, there are 16 purely bipartite networks, and 25 bipartite weighted networks, which yields 41 bipartite networks total (4% of the corpus).

Data sets that are multigraphs, but not multiplex/temporal or bipartite, are merely simplified by collapsing multi-edges. Edge weights are then discarded, and the resulting graph is processed starting from the check for directedness as above. In our corpus, there are 139 multigraphs and 2 weighted multigraphs, which yields 456 multigraphs total, including those that are multiplex.

Data sets that are only directed, with no other properties, are processed to produce three degree sequences: one each for the in-degrees, out-degrees, and total degrees. In our corpus, there are 103 purely directed networks (11.1% of data sets). In the case of a simple graph, the degree sequence is taken with no further processing. Our corpus

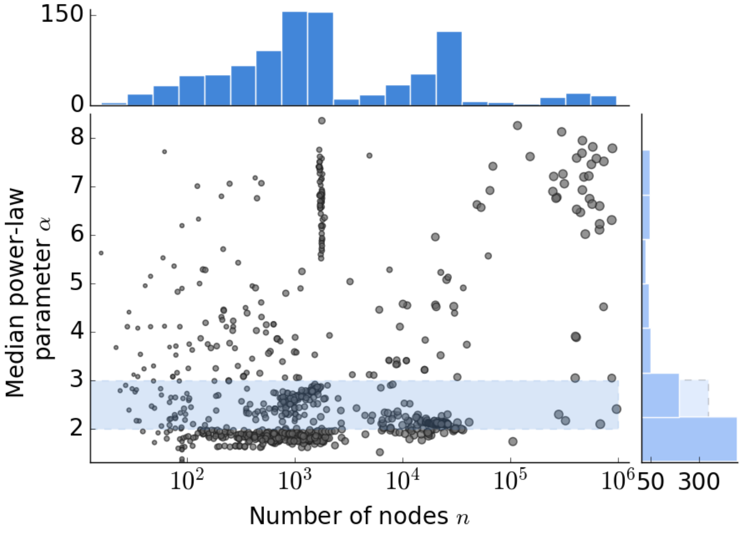

Supplementary Figure 3. **Median $\hat{\alpha}$ parameter versus network size n.** A horizontal band highlights the canonical $\alpha \in (2,3)$ range and illustrates the broad diversity of estimated power-law parameters across empiriworks.

contains 180 simple networks (19.4% of data sets).

Replication data, in the form of the corpus of degree sequences obtained by the above simplification steps, is available online (see main text). The corpus of 928 original network data sets represents approximately 250GB of data, and is hence not easily shareable; however, each network data set was publicly available at the time of writing, and could be found through the *Index of Complex Networks* at `icon.colorado.edu`.

Supplementary Note 2. Power-law analysis

1. *Fitting the model*

If the degree k follows a discrete power-law (scale-free) distribution starting at $k_{\min} \geq 1$, then pdf of the power law has the form

$$\Pr(k) = \frac{1}{\zeta(\alpha, k_{\min})} k^{-\alpha}$$

where $\zeta(\alpha, k_{\min}) = \sum_{i=0}^{\infty} (i + k_{\min})^{-\alpha}$ is the Hurwitz zeta function.

Estimating α requires first choosing $k_{\min}$, which we estimate via the standard Kolmogorov-Smirnov (KS) minimization approach [2]. This method selects the $k_{\min}$ that minimizes the maximum difference in absolute value between the (cumulative) empirical distribution $E(k)$ on the observed degrees $k \geq k_{\min}$ and the cdf of the best fitting power law $P(k \,|\, \hat{\alpha})$ on those same observations. This difference, called the KS statistic, is defined as

$$D = \max_{k \geq k_{\min}} |E(k) - P(k \,|\, \hat{\alpha})| \quad .$$

We choose as $k_{\min}$ the value that minimizes the D. The estimate $\hat{\alpha}$ is chosen by maximum likelihood (the MLE), which we obtain by numerically optimizing the log-likelihood function [2].

2. *Testing goodness-of-fit*

We assess the goodness-of-fit of the fitted model using a standard p-value, numerically estimated via the standard semi-parametric bootstrap approach [2]. Given a degree sequence with n elements, of which n_{tail} are $k \geq k_{\min}$ and with MLE $\hat{\alpha}$, a synthetic data set is generated as follows. For each of n synthetic values, with probability n_{tail}/n we

draw a random deviate from the fitted power-law model, with parameters $k_{\min}$ and $\hat{\alpha}$. Otherwise, we choose a value uniformly at random from the empirical set of degrees $k < k_{\min}$. Repeated n times this produces a synthetic data set that closely follows the empirical distribution below $k_{\min}$ and follows the fitted power-law model at and above $k_{\min}$.

Applying the previously defined fitting procedure to a large number of these synthetic data sets yields the null distribution of the KS-statistic $\Pr(D)$. Let D^* denote the value of the KS-statistic for the best fitting power-law model for the empirical degree sequence. The p-value for this model is defined as the probability of observing, under the null distribution, a KS-statistic at least as extreme as D^*. Hence, $p = \Pr(D \geq D^*)$ is the fraction of synthetic data sets with KS statistic larger than that of the empirical data set. Following standard practice for power-law degree distributions [2], if $p < 0.1$, then we reject the power law as a plausible model of the degree sequence, and if $p \geq 0.1$, then we fail to reject the model. We note: failing to reject does not imply that the model is correct, only that it is a plausible data generating process.

Supplementary Note 3. Alternative Distributions

1. *Exponential*

If k follows a discrete exponential distribution starting at $k_{\min}$, then the pdf of the exponential has the form

$$\Pr(k) = \left(\frac{\mathrm{e}^{-\lambda k_{\min}}}{1-\mathrm{e}^{-\lambda}}\right)\mathrm{e}^{-\lambda k} \ .$$

As with the power-law distribution, we use standard numerical maximization routines to estimate the maximum likelihood choice of λ.

2. *Log-normal*

The log-normal distribution is typically defined on a continuous variable k. To adapt this distribution to discrete values, we bin the continuous distribution and then adjust so that it begins at $k_{\min}$ rather than at 0.

Let $f(k)$ and $F(k)$ be the density and distribution functions of a continuous log-normal variable, where

$$f(k) = \frac{1}{\sqrt{2\pi}\sigma k}\mathrm{e}^{-\frac{(\log k-\mu)^2}{2\sigma^2}} \quad , \quad x > 0$$

and

$$F(k) = \frac{1}{2} + \frac{1}{2}\mathrm{erf}\left[\frac{(\log k - \mu)}{\sqrt{2}\sigma}\right] \ .$$

We define $g(k)$ and $G(k)$ to be the density and distribution functions of a discrete log-normal variable, given by

$$g(k) = F(k+1) - F(k) \quad , \quad x \geq 0$$

and

$$G(k) = \sum_{y=0}^{k} g(y) = F(k+1) - F(0) = F(k+1) \ .$$

We then generalize the distribution to start at some minimum value, i.e., rather than starting at 0, the distribution starts at $k = k_{\min}$, where $k_{\min}$ is a positive integer. This pmf is obtained by re-normalizing the tail of $g(k)$ so that it sums to 1 on the interval $k_{\min}$ to ∞, yielding

$$\begin{aligned} h(k) &= \frac{g(k)}{\sum_{k=k_{\min}}^{\infty} g(k)} = \frac{g(k)}{1-\sum_{k=0}^{k_{\min}-1} g(k)} \\ &= \frac{g(k)}{1-G(k_{\min}-1)} = \frac{g(k)}{1-F(k_{\min})} \ . \end{aligned}$$

Maximum likelihood estimation was carried out using standard numerical optimization routines. Additionally, we constrained the optimization in order to prevent numerical instabilities. Specifically, we required $\sigma \geq 1$ and $\mu \geq -\lfloor n/5 \rfloor$. As a check on these constraints, we verified that in no cases did the likelihood improve significantly by allowing $\sigma < 1$, and the constraint on μ prevents it from decreasing without bound (a behavior that can produce arbitrarily heavy-tailed distributions over a finite range in the upper tail). To initialize the numerical search, we set $(\mu_0, \sigma_0) = (0, 1)$.

3. *Power-law with exponential cutoff*

If k follows a discrete power-law distribution starting at $k_{\min}$, and with an exponential cutoff in the upper tail, then its pdf has the form

$$\Pr(k) = \left[\mathrm{e}^{-k_{\min}\lambda}\,\Phi(\mathrm{e}^{-\lambda}, \alpha, k_{\min})\right] k^{-\alpha}\mathrm{e}^{-\lambda k}$$

where $\Phi(z, s, a) = \sum_{i=0}^{\infty} \frac{z^i}{(a+i)^s}$ is the Lerch Phi function. We estimate this distribution's parameters λ and α using standard numerical maximization routines.

4. *Weibull (Stretched exponential)*

A common approach to obtain a discrete version of the stretched exponential or Weibull distribution is to bin the continuous distribution [3]. Let $f(k)$ and $F(k)$ be the density and distribution functions of a continuous Weibull variable, where

$$F(k) = 1 - \mathrm{e}^{-(k/b)^a}, \quad x \geq 0 \ .$$

Define $g(k)$ and $G(k)$ to be the density and distribution functions of a discrete Weibull variable, given by:

$$g(k) = F(k+1) - F(k), \quad x \geq 0$$

and

$$G(k) = \sum_{y=0}^{k} g(y) = F(k+1) - F(0) = F(k+1) \ .$$

As with the log-normal, we generalize the distribution to start at some minimum value, i.e., rather than starting at 0, the distribution starts at $k = k_{\min}$, where $k_{\min}$ is a positive integer. This pmf is obtained by re-normalizing the tail of $g(k)$ so that it sums to 1 on the interval $k_{\min}$ to ∞, yielding

$$h(k) = \mathrm{e}^{(k_{\min}/b)^a}\left[\mathrm{e}^{-(k/b)^a} - \mathrm{e}^{-((k+1)/b)^a}\right] \ .$$

We estimate this distribution's parameters using standard numerical maximization routines.

Supplementary Note 4. Likelihood-ratio tests

In the primary evaluation, the power-law models were compared with the alternatives using a set of likelihood ratio tests. These likelihood ratio tests have been previously shown valid for both the nested and non-nested models considered here [2, 4], and have lower incorrect decision rates [2] compared to simple penalized likelihood approaches to model comparison. In Supplementary Note 5, we describe an alternative evaluation that uses information criteria [5] in place of the likelihood ratio test.

For each alternative distribution, we obtained the log-likelihood $\mathcal{L}_{\mathrm{Alt}}$ of the best fit. The difference between this value and the log-likelihood of the power-law fit to the same observations yields the likelihood ratio test (LRT) statistic $\mathcal{R} = \mathcal{L}_{\mathrm{PL}} - \mathcal{L}_{\mathrm{Alt}}$. When $\mathcal{R} > 0$, the power law is a better fit to the data, and when $\mathcal{R} < 0$, the alternative distribution is the better fitting model. Crucially, when $\mathcal{R} = 0$, the test is inconclusive, meaning that the data cannot distinguish between the two models.

The test statistic $\mathcal{R}$, however, is itself a random variable, and hence is subject to statistical fluctuations. Accounting for these fluctuations dramatically improves the accuracy of the test by reducing both types of incorrect decision rates [2]. As a result, the sign of $\mathcal{R}$ alone is not a reliable indicator of which model is a better fit. The now standard approach for controlling for this uncertainty is to calculate a p-value against the null model of $\mathcal{R} = 0$, under a two-tailed null hypothesis test. Only when that model can be rejected is the sign of $\mathcal{R}$ meaningful [4]. In this setting, if $p < 0.1$, then the absolute value of $\mathcal{R}$ is sufficiently far from 0 that its sign is interpretable.

We obtain this p-value with the same method used in Ref. [2], originally proved valid in Ref. [4]. Note that

$$\begin{aligned}\mathcal{R} &= \mathcal{L}_{\mathrm{PL}} - \mathcal{L}_{\mathrm{Alt}} \\ &= \sum_{i=1}^{n} \left[\ln \Pr_{\mathrm{PL}}(k_i) - \ln \Pr_{\mathrm{Alt}}(k_i)\right] \\ &= \sum_{i=1}^{n} \left[\ell_i^{(\mathrm{PL})} - \ell_i^{(\mathrm{Alt})}\right]\end{aligned}$$

where $\ell_i^{(\mathrm{PL})}$ is the log-likelihood of a single observed degree value k_i under the power-law model, and n is the number of empirical observations being used by a model (in our setting, this number is n_{tail}, but we omit that annotation to keep the mathematics more compact).

We have assumed that the degree values k_i are independent, which means the point-wise log-likelihood ratios $\ell_i^{(\mathrm{PL})} - \ell_i^{(\mathrm{Alt})}$ are independent as well. The central limit theorem states that the sum of independent random variables becomes approximately normally distributed as their number grows large, and that this normal distribution has mean μ and variance $n\sigma^2$, where σ^2 is the variance of a single term. This distribution can be used to obtain the p-value, but requires that we first estimate μ and σ^2. Note that we assume $\mu = 0$ because the null hypothesis is $\mathcal{R} = 0$. We then approximate σ^2 as the sample variance in the observed $\mathcal{R}$

$$\sigma^2 = \frac{1}{n-1} \sum_{i=1}^{n} \left[\left(\ell_i^{(\mathrm{PL})} - \ell_i^{(\mathrm{Alt})}\right) - \left(\bar{\ell}_i^{(\mathrm{PL})} - \bar{\ell}_i^{(\mathrm{Alt})}\right)\right]^2 \quad ,$$

where

$$\bar{\ell}_i^{(\mathrm{PL})} = \frac{1}{n} \sum_{i=1}^{n} l_i^{(\mathrm{PL})} \quad \text{and} \quad \bar{\ell}_i^{(\mathrm{Alt})} = \frac{1}{n} \sum_{i=1}^{n} \ell_i^{(\mathrm{Alt})}$$

are sample means.

Under this null distribution, the probability of observing an absolute value of $\mathcal{R}$ at least as large as the actual test statistic is given by the two-tail probability

$$p = \frac{1}{\sqrt{2\pi n\sigma^2}} \left[\int_{-\infty}^{-|\mathcal{R}|} \mathrm{e}^{-\frac{t^2}{2n\sigma^2}}\,\mathrm{d}t + \int_{|\mathcal{R}|}^{\infty} \mathrm{e}^{-\frac{t^2}{2n\sigma^2}}\,\mathrm{d}t\right] . \tag{1}$$

Hence, following standard practice [2], if $p \leq 0.1$, then we reject the null hypothesis that $\mathcal{R} = 0$, and proceed by interpreting the sign of $\mathcal{R}$ as evidence in favor of one or the other model.

SUPPLEMENTARY DISCUSSION

Supplementary Note 5. Robustness checks

1. Results for simple networks alone

Extending the scale-free network hypothesis to apply to networks that are not naturally simple allowed us to draw on a much larger range of empirical network data sets. It is therefore possible that the non-simple network data sets present in the corpus have structural patterns distinct from those of simple networks, and hence are less likely to exhibit a scale-free pattern. We test for this possibility by examining the classifications of the 180 simple networks within the corpus. Among these networks, a minority exhibit neither direct nor indirect evidence of scale-free structure (53% Not Scale Free), and a modest majority exhibit at least indirect evidence (40% Super-Weak; Supplementary Figure 4). Compared to the overall corpus, there is a notable increase in the Weakest and Weak categories. These

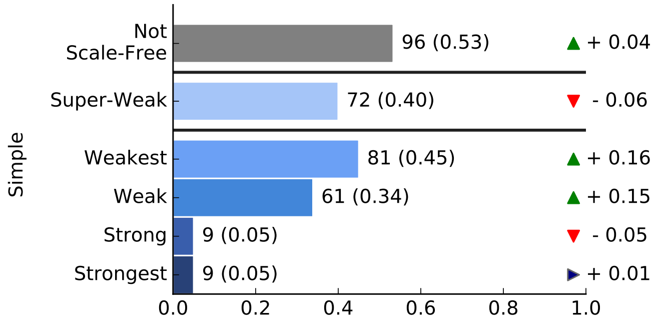

Supplementary Figure 4. Proportions of networks in each scale-free evidence category for simple networks.

differences can be partly explained by the distribution of simple graphs by domain, as 72% of simple graphs in the corpus are social, which exhibits similar proportions across the evidence categories. Hence, the structural diversity of real-world networks observed for the corpus as a whole is also observed when we restrict our analysis to only simple graphs, and neither our inclusion of non-simple graphs, nor the graph simplification procedure described above, have skewed our results.

2. *Results after removing power law with exponential cutoff from alternatives*

To rule out potential bias against the scale-free hypothesis as a result of the inclusion of a power-law-like alternative in the Strong and Strongest evidence categories, we also examine the results when we remove the power law with exponential cutoff from our list of alternative distributions. As the power law is a special case of the power law with cutoff, our likelihood-ratio test can only be inconclusive or result in favor of the power law with cutoff. In the case where the power law with cutoff is the best model, this case cannot be placed in the Strongest or Strong scale-free categories by definition. In our primary evaluation, 9.59% of data sets fall into the Strong category. When we include data sets for which the power law with exponential cutoff was favored over the power law, this increases negligibly to 10.4% of data sets.

Additionally, if we also remove the restriction on the range of $\hat{\alpha}$, the percentage of data sets in this Strong category increases to 18%. This is very close to the results for the Weak category (19%), which indicates that the majority of the decrease from the Weak to the Strong is due to the imposition of the bounds on $\hat{\alpha}$ rather than the requirement against favoring alternative distributions.

There is a similarly negligible increase in the number of data sets in the Strongest category, from 3.88% to 4.63% when we allow data sets for which the power law with exponential cutoff is favored. This shift is consistent with the fact that the construction of our likelihood ratio test favors the power-law distribution since all alternatives inherit the $k_{\min}$ that maximizes the likelihood of the power-law fit, rather than choosing their own best-fitting value.

3. *Results for directed networks alone, after removing percent constraints*

Because directed networks are often a specific focus within the scale-free literature, we also examine the results for the 103 directed networks in our corpus, under the "maximally permissive" alternative parameterization of the evidence categories (see main text). That is, we consider their classification when we require only one of the associated degree sequences to satisfy the requirements of a particular evidence category.

We find that the distribution of these data sets across the evidence categories (Supplementary Figure 5) is very close to the results over the entire corpus, implying that our evaluation scheme is not biased against directed scale-free networks.

4. *Results for the largest connected components alone*

The graph simplification process described above, and used in the primary evaluation, considers all components in a given graph. As an alternative specification, we consider a check for connectedness of a network: If a network is not

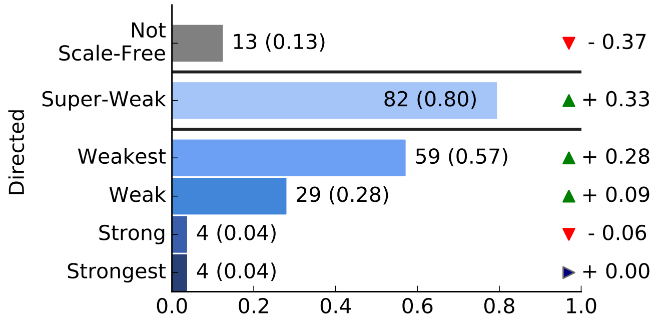

Supplementary Figure 5. Proportions of networks in each scale-free evidence category for directed networks with removed degree percentage requirements.

connected (i.e., it contains more than one component), we extract two degree sequences, one for the largest connected component, and one for the entire graph.

Including degree sequences for each largest connected in a network data set produces quantitatively similar results as when excluding it, and the overall conclusions remain unchanged. The proportion of networks in each scale-free category differs by at most 6% from the results in the main text.

5. *Results for scaling behavior of degree heterogeneity*

In addition to the degree heterogeneity analysis described in the main text, we consider a second test using the naturally simple networks, which are characterized by a single degree sequence. Given the fitted power-law distribution for each such network, we generated synthetic networks whose degree distribution is given by a semi-parametric model: the degrees below $k_{\min}$ are given by the empirical frequencies, while the degrees at and above $k_{\min}$ are given by the fitted power-law distribution. Hence, these synthetic networks are scale-free networks, by construction. For each simple network in this set, we generated 12 synthetic networks and compared the degree heterogeneity statistic $\langle k^2 \rangle / \langle k \rangle^2$ as a function of n for the empirical and synthetic degree distributions.

The synthetic networks, especially at larger sizes, tend to have a larger variance than the empirical distributions (Supplementary Fig. 8), indicating that the empirical networks have substantially less degree heterogeneity than would be predicted if they were, in fact, scale free. That is, the scaling of these empirical moment ratios is not diverging as quickly as predicted by the scale-free hypothesis.

6. *Results of model comparisons using information criteria*

Information criteria are a common approach for selecting the best model from among a set of fitted models [5]. As an alternative to the normalized likelihood ratio test approach we use in our primary evaluation scheme, we now describe and apply an alternative model comparison method based on replacing the LRT with an application of the Akaike information criterion (AIC).

Under the AIC, a model's adjusted "score" is written as $2k - 2\log\mathcal{L}$, where k is the number of model parameters and $\mathcal{L}$ is the model's likelihood when fitted to the data. The power-law distribution used here is considered to have two estimated parameters: one in the form of α, the scaling exponent, and one in the form of the minimum value $k_{\min}$, which determines the left truncation of the degree sequence to be fitted. Because all alternative distributions in our comparison inherit the value of $k_{\min}$ from the fitted power law, this minimum value is not considered a parameter for them. Hence, all alternative distributions have exactly two parameters, except for the exponential, which has one.

The Bayesian information criterion (BIC) (sometimes called the Schwarz criterion) is another commonly used method to compare models, but it offers little utility over the AIC in the particular setting considered here. The BIC score is written as $k\log n - 2\log\mathcal{L}$, where n is the number of observations fitted by the model. Hence, the BIC imposes a stronger, sample-size-dependent separation between models with different complexities (number of parameters) compared to AIC. However, because all distribution models considered in our evaluation have exactly two parameters, except for the exponential which has one, the BIC will offer little insight beyond what is already provided by the AIC. For this reason, we focus our analysis on the AIC and mention results for the BIC when relevant.

Alternative	$p(x) \propto f(x)$	$M_{\rm PL}$	Test Outcome Inconclusive	$M_{\rm Alt}$
Exponential	$\mathrm{e}^{-\lambda x}$	**36%**	13%	51%
Log-normal	$\frac{1}{x}\,\mathrm{e}^{-\frac{(\log x-\mu)^2}{2\sigma^2}}$	**14%**	31%	55%
Weibull	$\mathrm{e}^{-\left(\frac{x}{b}\right)^a}$	**37%**	13%	50%
Power law with cutoff	$x^{-\alpha}\mathrm{e}^{-\lambda x}$	**0**	42%	58%

Supplementary Table II. Comparison of scale-free and alternative distributions, using AIC. The percentage of network data sets that favor the power-law model $M_{\rm PL}$, alternative model $M_{\rm Alt}$, or neither, under a standard AIC comparison (see text), along with the form of the alternative distribution $f(x)$.

For each degree sequence, we compare the power-law model's AIC score with the AIC score of each alternative distribution, deriving ΔAIC. Following standard practice, if $\Delta\text{AIC} < 2$, we conclude that there is little or no statistical evidence that the models fit the data differently [6]. In this case, we say that the comparison is inconclusive and cannot distinguish between the two models. (This outcome is comparable to failing to reject the null of $\mathcal{R} = 0$ in the normalized LRT.) Otherwise, when $\Delta\text{AIC} \geq 2$, we conclude that the model with the lower AIC value provides the better fit to the data.

Under the AIC approach to comparing models, the percentages of network data sets that either favor the power-law model, favor the alternative model, or are inconclusive (Supplementary Table II) are very close to those produced under the normalized LRT used in the primary evaluation. In fact, we note that the results are slightly more more in favor of each alternative distribution under the AIC than under the LRT. Using the BIC instead of the AIC produces identical percentages for all distributions except the exponential, as explained above. The BIC results favor the exponential distribution more strongly than the AIC, in which only 16% of data sets favor the power-law model under the BIC, while 77% favor the exponential. For categorizing data sets according to their levels of evidence for scale-free structure, we only used the AIC below, as using the BIC would not change our conclusions.

In order to use an information criterion to make the model comparisons necessary to categorize a data set, we replace the LRT comparison with an AIC-based comparison, following the AIC rules stated in the preceding paragraph for concluding whether one distribution or another is favored. In this way, the category definitions themselves, and hence their interpretation, do not change, and we have only changed the method by which we decide whether an alternative distribution is favored over the power law. For succinctness, we repeat, without modification, the text of those definitions here:

**Super-Weak**	For at least 50% of graphs, no alternative distribution is favored over the power law.
**Weakest**	For at least 50% of graphs, a power-law distribution cannot be rejected ($p \geq 0.1$).
**Weak**	Requirements of *Weakest*, and the power-law region contains at least 50 nodes ($n_{\rm tail} \geq 50$).
**Strong**	Requirements of *Weak* and *Super-Weak*, and $2 < \hat{\alpha} < 3$ for at least 50% of graphs.
**Strongest**	Requirements of *Strong* for at least 90% of graphs, and requirements of *Super-Weak* for at least 95% of graphs.
**Not Scale-Free**	Networks that are neither Super-Weak nor Weakest.

We note that the percentage thresholds given in the Strongest category were chosen to match the expected error rates of the LRT. While there is no equivalent expectation for the AIC, we retain these thresholds for the sake of consistency and ease of comparison with the results of our primary evaluation.

Under this AIC-based evaluation, we find that the proportion of networks in each scale-free evidence category is nearly identical to the results produced using likelihood ratio tests (Supplementary Fig. 6). This robustness indicates that our conclusions are not driven by the assumptions of the particular method by which we compare alternative distributions to the power-law model. Moreover, applying the AIC-based evaluation to only the simple networks, as a further robustness check, produces nearly identical results to that of using the likelihood ratio tests (Supplementary Fig. 7), again indicating that our conclusions are robust to variations in how models are compared.

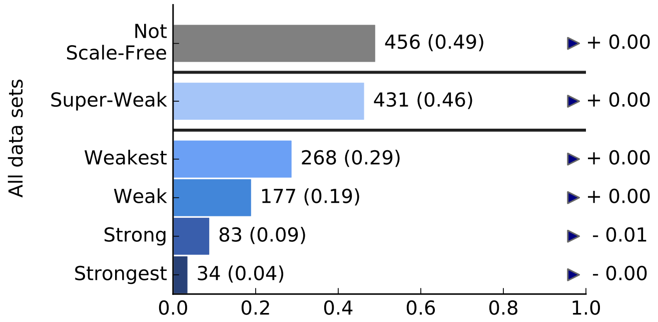

Supplementary Figure 6. Proportions of networks in each scale-free evidence category using AIC instead of LRT for comparison of alternative distributions. Tickers indicate percent change from the results in the main text.

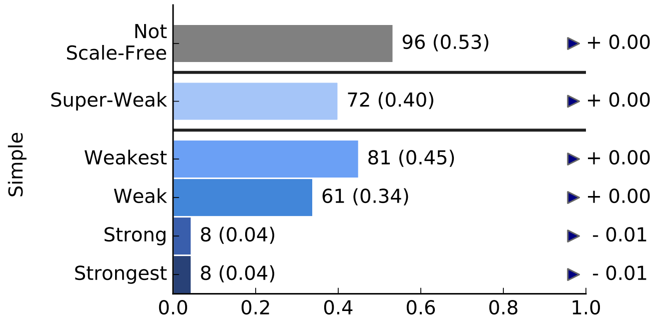

Supplementary Figure 7. Proportions of simple networks in each scale-free evidence category using AIC instead of LRT for comparison of alternative distributions. Tickers indicate percent change from the results for simple networks in the main text.

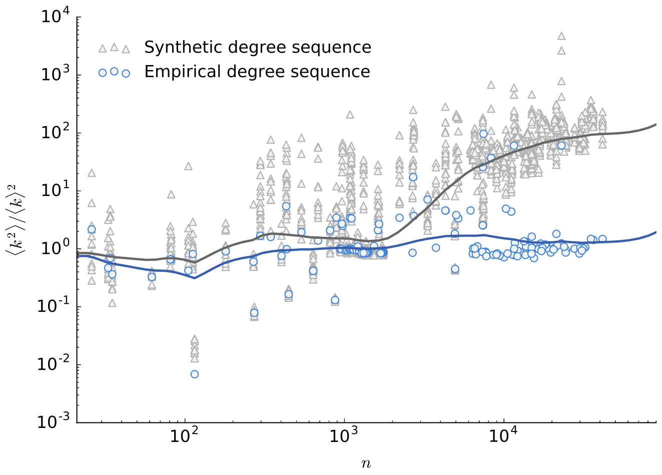

Supplementary Figure 8. Scatterplot of the degree heterogeneity factor for empirical and synthetic simple networks vs their size. Blue points are empirical networks and 12 synthetic networks were generated from the best power-law fit for each, shown in grey.

Supplementary Note 6. Evaluating the method on synthetic data with ground truth

To test the accuracy of the evaluation scheme, we tested it on four types of synthetic network data sets with known ground truth structure. For each type, we conducted a numerical experiment using 100 instances of $n = 5000$ node network data sets. Three of these types generate scale-free structure by design: (i) one generated by a simple version of linear preferential attachment [7], (ii) one by a simple vertex copying model [8], and (iii) one by the configuration

model [9] to create a temporal network where every snapshot is scale free (with $n = 1000$ nodes). The fourth type generates non-scale-free networks by design, (iv) using Erdős-Rényi random graphs.

The first type of synthetic network is generated by a simple version of linear preferential attachment [7], which is one of the most commonly referenced mechanisms for generating scale-free networks. The process is as follows, and results in a directed, unweighted, connected network. The assembly process begins with a $n = 4$ node directed network, in which each node has $k^{(\text{out})} = 3$ out edges, one to each of the other nodes. We then add one node at a time until we reach a total of $n = 5000$ nodes in the network. Each added node forms $k^{(\text{out})} = 3$ out edges. For each out edge, with probability $p = 2/3$ the connection is formed preferentially, i.e., the new node i connects to an existing node j with probability proportional to j's in-degree $k_j^{(\text{in})}$. Otherwise, the connection is formed uniformly, i.e., the new node i connects to an existing node j with constant probability. The in-degrees distribution of the final network is scale free, following a power law of the form $k^{-2.5}$, while the out-degree distribution is a delta function at $k = 3$. The graph simplification procedure takes this directed network and produces three degree sequences, corresponding to the in-, out-, and total degrees. The in- and total degree sequences have power-law tails (the total degree sequence follows a power law for $k \gg 3$). Hence, we would expect these networks to fall into the Strong category because 2 of the 3 degree sequences are scale free.

Under our primary evaluation scheme, with thresholds set as described in the main text, we find that 89% of the synthetic networks assembled by this simple model of linear preferential attachment fall into the Super-Weak category. Omitting the power law with cutoff as an alternative model increases this rate to 97%, meaning that only 3% of the time, some alternative is a better fit to the data than is a scale-free distribution. Considering the plausibility of the fitted power laws, we find that 54% of these networks fall into the Weakest and Weak categories, 52% in the Strong category, and none in the Strongest category. As expected, the in-degree sequences and total degree sequences are generally plausible power laws (80% and 67%, respectively), while the out-degree sequences never are. The modest deviations of the plausibility rates for the in- and total degree sequences from the expected rate of 90% (which is set by the choice of critical threshold for the null hypothesis test) are likely attributable to finite-size effects.

The absence of these networks in the Strongest category is entirely due to the fact that this category requires that 90% of associated simple graphs be plausibly power law, while theoretically, only 67% (2 of 3) of the simple graphs can be. While it may seem counter-intuitive to some that preferential attachment networks, a canonical example of a scale-free network in the literature, do not fall into the Strongest category, this result is by construction because every associated degree sequence is given an equal weight in the classification scheme. However, under the maximally permissive parameterization of the evaluation scheme, in which we relax the threshold requirements to allow inclusion in a category if even one degree sequence meets the requirements, i.e., if either the in-, out-, or total degree sequences are plausibly scale free, then 93% of preferential attachment networks fall into the Strongest category.

The second type of synthetic network is generated by a simple vertex-copying model [8], and also produces scale-free structure. The process is as follows, and results in an directed, unweighted, connected network. The assembly process begins with a $n = 4$ node directed network, in which each node has $k^{(\text{out})} = 3$ out edges, one to each of the other nodes. We then add one node at a time until we reach a total of $n = 5000$ nodes in the network. For each new node v we add, we first pick an existing node u uniformly at random. Then, for each edge (u, w), we add an edge (v, w) with probability $q = 0.6$, i.e., v copies u's link to w. Otherwise, we choose a uniformly random node x and add the edge (v, x), i.e., v choose a uniformly random node to link to. This process is repeatedfor each of the $k_v^{(\text{out})} = 3$ outgoing edges u has. The in-degree distributions of the final network is scale free, following a power lawof the form $k^{-\alpha}$, with $\alpha = 1 + \frac{1}{q} = 2.67$, while the out-degree distribution is a delta function at $k = 3$. The graph simplification procedure takes this directed network and produces three degree sequences, corresponding to the in-, out-, and total degrees. The total-degree distribution looks like $k^{(\text{in})} + k^{(\text{out})} = k^{-2.67} + 3 \approx k^{-2.67}$for $k \gg 3$ Hence, we would expect these networks to fall into the Strong category because 2 of the 3 degree sequences are scale free.

Under the primary evaluation scheme, with thresholds set as described in the main text, we find that 83% of these synthetic networks graphs fall into the Super-Weak category. Omitting the power law with cutoff as an alternative increases this rate to 97%. Furthermore, we find that 72% fall into the Weakest and Weak categories, meaning the power law is plausible with at least 50 points in the tail of the degree sequence, and 68% fall into the Strong category and none in the Strongest category. Because only 2 of the 3 degree distributions have power-law tails, the same reasoning for preferential attachment networks applies here. And, under the maximally permissive parameterization of the evaluation scheme, we find that 97% of these networks fall into the Strongest category.

The third type of synthetic network is generated using the configuration model [9], and produces a network that is expected to fall into the Strongest category, i.e., a network where every associated degree sequence is scale free. Toward this end, we construct a temporal network, where each of $T = 20$ layers has a degree sequence of $n = 1000$ nodes drawn iid from a power-law distribution with $\alpha = 2.5$. To connect the nodes in a given layer, we use the Havel-Hakimi algorithm [10, 11] to generate an initial condition for a degree-preserving edge-swapping algorithm that can sample uniformly at random from the set of simple graphs with the specified degree sequence [9].

Under the primary evaluation scheme, with thresholds set as described in the main text, we find that 100% of these synthetic networks fall into the Super-Weak, Weakest, Weak, and Strong categories, and 59% fall into the Strongest category. This latter rate falls below the expected rate, likely because of finite-size effects. Under the the maximally permissive parameterization of the evaluation scheme, 100% of these networks fall into the Strongest category.

The fourth type of synthetic network is a simple Erdős-Rényi random graph $G(n, p)$, which has no scale-free structure. In these networks, each edge exists iid with probability $p = c/(n-1)$, where c is the mean degree. To ensure that these networks are sparse and are largely connected, we set $c = 6$. For this choice, the degree distribution is Poisson with mean c, which has a "thin" or light tail, compared to the power law.

Under the primary evaluation scheme, with thresholds set as described in the main text, we find that only 15% are classified as even Super-Weak, although this rate increases to 26% if the power law with cutoff is omitted as an alternative. Furthermore, we find that 42% and 40% of these networks fall into the Weakest and Weak categories, respectively. The fitted power-law distributions for these networks all have very large scaling parameters (the smallest is $\hat{\alpha} = 6.36$), reflecting the thin-tailed structure of their degree distributions, and hence none are classified as falling into the Strong or Strongest categories. This behavior highlights the fact that a network falling into the Weakest or Weak categories can be indicative of the power-law estimation routines finding some marginal part of the extreme upper tail that is plausibly power-law distributed, even when the underlying distribution is not scale free. As $G(n, p)$ random graphs are simple, the above results are unchanged under the maximally permissive parameterization of the evaluation scheme.

Supplementary References

[1] A. Clauset, E. Tucker, M. Sainz (2016). The Colorado Index of Complex Networks, `icon.colorado.edu`.

[2] A. Clauset, C. R. Shalizi, M. E. J. Newman, Power-Law Distributions in Empirical Data. *SIAM Review* **51**, 661–703 (2009).

[3] T. Nakagawa, S. Osaki, The discrete weibull distribution. *IEEE Trans. Reliability* **24**, 300–301 (1975).

[4] Q. H. Vuong, Likelihood Ratio Tests for Model Selection and Non-Nested Hypotheses. *Econometrica* **57**, 307–333 (1989).

[5] G. Claeskens, N. L. Hjort, *Model Selection and Model Averaging* (Cambridge University Press, Cambridge, England, 2008).

[6] K. P. Burnham, D. R. Anderson, *Model selection and multimodel inference: a practical information-theoretic approach* (Springer-Verlag, 2002).

[7] D. Easley, J. Kleinberg, *Networks, Crowds, and Markets: Reasoning about a Highly Connected World* (Cambridge University Press, 2010).

[8] M. Newman, *Networks: An Introduction* (Oxford Univerity Press, 2010).

[9] B. K. Fosdick, D. B. Larremore, J. Nishimura, J. Ugander, Configuring Random Graph Models with Fixed Degree Sequences. *SIAM Rev.* **2**, 315-355 (2017).

[10] V. J. Havel, A remark on the existence of finite graphs. *Casopis Pest. Mat,* **1253**, 477-480 (1955).

[11] S. L. Hakimi, A remark on the existence of finite graphs. *Journal of the Society for Industrial & Applied Mathematics* **1**, 135-147 (1963).
